# Association between psychosocial stressors and temporomandibular disorders in clinical dental students: a cross-sectional study

**DOI:** 10.7717/peerj.19066

**Published:** 2025-02-27

**Authors:** Abdullah Alqarni, Jagadish Hosmani, Saeed Alassiri, Ali Mosfer A. Alqahtani, Ali Alfaifi, Shuayl Abdulaziz Al Jazea

**Affiliations:** 1 Department of Diagnostic Science and Oral Biology, College of Dentistry, King Khalid University, Abha, Saudi Arabia; 2 Department of Prosthodontics, College of Dentistry, King Khalid University, Abha, Saudi Arabia; 3 Department of Surgery, The British University in Egypt, El Sherouk City, Egypt

**Keywords:** Temporomandibular joint disorders, Anxiety, Dental students, Oral health, Psychological distress

## Abstract

**Background:**

Temporomandibular disorders (TMDs) comprise an extensive spectrum of conditions that originate from diverse complex components of the temporomandibular joint. It is generally acknowledged that the biopsychosocial model is the preeminent framework for understanding the aetiology of TMDs. Anxiety, depression, and tension are among the psychological disorders that are commonly observed in dental students. The current research probed the propinquity of psychosocial stressors and TMD-like symptoms among clinical dental undergraduates residing in the Aseer region of Saudi Arabia, with a specific emphasis on the functional implications for their overall well-being.

**Methods:**

This research included 89 clinical dentistry students who completed online questionnaires. The Patient-Reported Outcomes Measurement Information System (PROMIS) and Oral Health Impact Profile for Temporomandibular Disorders (OHIP-TMD) were used in these surveys. The trait-related attributes of TMD impacting the oral health profile were assessed using principal component analysis. Demographic factors for anxiety and TMD were examined using linear regression. The psychosocial and functional variables of the OHIP-TMD were compared with those of anxiety in the general regression system.

**Results:**

The mean Oral Health Impact Profile (OHIP) value was 0.60, with a standard deviation of 0.61, indicating that the majority of respondents reported no or infrequent impacts on their oral health profile. Gender was a significant predictor of OHIP scores (*P* < 0.05), with females reporting higher scores. Despite these higher scores among females, the overall impact on oral health remained minimal for most respondents. The average PROMIS score was 11.12, with a standard deviation of 3.84. The PROMIS regression analysis on demographic variables yielded an R^2^ value of 0.092, F(4,78) = 5.691, with significance at *P* < 0.05. Gender emerged as the most significant predictor of PROMIS scores (*P* < 0.05), with females reporting higher scores. Once again, despite the higher scores among females, the overall impact remained low for most respondents. An analysis using a general linear model revealed a significant correlation between heightened anxiety levels and an increase in both psychosocial problems and physical function.

**Conclusion:**

Although the majority of respondents reported minimal impacts on their oral health, anxiety remained a significant issue among female clinical dentistry students at the College of Dentistry in Aseer Province, Kingdom of Saudi Arabia. This anxiety was closely linked with psychological distress and impaired oral physical function. Beyond the realm of dental health, anxiety also had a profound effect on academic performance and student engagement. Therefore, addressing student anxiety is essential for enhancing overall well-being and academic success.

## Background

Temporomandibular disorders (TMDs) encompass a range of conditions affecting the temporomandibular joint (TMJ), masticatory muscles, and associated structures. These disorders can present through various physical symptoms, including orofacial pain, joint discomfort, and restricted jaw mobility. Increasingly, psychological factors, such as anxiety and stress, have been shown to contribute to TMD symptomatology by exacerbating muscle tension and pain sensitivity (de Leeuw & Klasser, 2015). TMDs are often classified into subgroups based on their origin: either articular or muscular. In the first situation, symptoms are specifically related to the TMJ; in contrast to the subsequent case, symptoms are specifically linked to the stomatognathic anatomy (Valesan et al., 2021).

The etiology of TMD is difficult to recognize, although it results from a combination of psychological, physiological, structural (occlusion and trauma), and postural variables (parafunctional habits) and genetic components. These situations have the potential to disturb the equilibrium of the stomatognathic system, leading to the emergence of symptoms and issues associated with TMDs (Resende et al., 2020). According to a recent investigation, the prevalence of TMDs was found to be approximately 31% in adults and elderly individuals and approximately 11% in children and adolescents (Chisnoiu et al., 2015). A reduction in disc displacement was the most common subtype of TMD, occurring in close to 26% of the aged individuals and 7.5% of the juveniles (Minervini et al., 2023). Commonly reported symptoms include pain in the TMJ, auricles, champing musculature, faculty of sight, and countenance; emotional stress; limited physical function; joint noises or locking; and restricted jaw movements (Chisnoiu et al., 2015; Minervini et al., 2023).

Prior research has underscored the influential role of psychosocial factors in the development of TMD, demonstrating a higher occurrence among TMD patients compared to healthy individuals. TMJ disorders are often associated with anxiety, which can impact pain perception and muscle over activity. This can be classified as state anxiety, which results from a particular stressor, or trait anxiety, which is a lasting behavioral characteristic (De La Torre Canales et al., 2018; Häggman-Henrikson et al., 2020; Simoen et al., 2020).

According to a recent analysis, people with myofascial pain experience increased levels of anxiety and depression. When evaluating patients with TMD, it is essential to consider the biopsychosocial components separately (Reis et al., 2022).

Oral health-related quality of life (OHRQoL) involves evaluating a patient’s functional abilities (such as munching, napping, and withering), sagacity, and delectation with oral health care (Raghavendra et al., 2007; Sischo & Broder, 2011). Comprehending a patient’s OHRQoL enables a more individualized approach to their care beyond mere medical-dental interventions. Research has shown that people with TMD have more difficulties with their OHRQoL than do the silent majority. The presence of symptoms such as periarticular pain and restricted jaw movement significantly influences the socioeconomic status of patients (Pallegama et al., 2005; Reissmann et al., 2018).

Several studies have shown that anxiety augments the risk of developing TMD. Given the high levels of anxiety experienced by students during their university studies, it is logical to anticipate an increased susceptibility to developing TMD as a result of this anxiety link (Theroux et al., 2019; Akhter et al., 2019; Luo et al., 2023).

Dental students are a high-stress population, making them an ideal cohort for investigating anxiety-related TMD symptoms. While TMD symptoms may not always reach clinical thresholds in this group, subclinical presentations can provide valuable insights into early psychosocial drivers of TMD (Srivastava et al., 2021; Alamri et al., 2020; Fernandes Azevedo et al., 2017; AlHussaini et al., 2019). Investigating the impact of TMD corollaries on the wellbeing of dental students and how anxiety plays a role could lead to the development of intercessions to grapple with these quandaries. Nevertheless, there is a scarcity of studies regarding anxiety and TMD among dental students in Aseer Province in the Kingdom of Saudi Arabia.

Recent studies indicate a notable prevalence of both stress and TMD among populations across various regions in Saudi Arabia. For instance, TMD prevalence among dental students in Riyadh was reported at 47%, with stress cited as a primary influencing factor (AlHussaini et al., 2019). Additionally, in a study of Saudi university students, approximately 65% reported moderate to high stress levels, with academic and social pressures being the main contributors (Al-Khotani et al., 2016). A study examined the prevalence of TMD among university students in North Saudi Arabia. It found that 49.7% of participants exhibited at least one TMD symptom, with clicking and periauricular pain being the most common. The study highlighted higher rates in females and students from science/health faculties, suggesting a possible link to stress and study demands (Zwiri & Al-Omiri, 2016). Another study assessed the prevalence of temporomandibular joint dysfunction (TMD) among edentulous patients in Saudi Arabia and found that 60.5% showed TMD signs, with higher reports in females (63.12%) compared to males (58.75%). The most common symptom was limited mouth opening, while joint noises like clicking were least common. The findings were not significantly linked to the duration of being edentulous (AlZarea, 2017). Other studies show that in general Saudi populations, TMD prevalence ranges between 25% and 40%, with stress levels significantly correlated with increased TMD symptoms (Nadershah, 2019). These findings highlight a considerable frequency of stress-induced TMD symptoms within Saudi populations, reinforcing the need to explore these associations specifically in high-stress environments like dental training.

This study investigates the association between psychosocial stressors, specifically anxiety, and TMD-like symptoms in clinical dental students. By focusing on this high-stress group, the study aims to shed light on the impact of psychological stress on TMD-like symptoms and OHRQoL, emphasizing the need for targeted interventions to alleviate stress-related orofacial discomfort in this population.

The rationale for this design lies in established research showing that clinical dental students, due to high exposure to stress and anxiety, are more likely to experience orofacial symptoms consistent with TMD, even if subclinical. This study leverages the OHIP-TMD scale to quantify the impact of these symptoms on OHRQoL and provides insights into how anxiety, in particular, influences symptom prevalence and severity. As such, we used a validated tool (OHIP-TMD) that captures psychosocial impact while acknowledging that not all orofacial pain in this cohort is clinically attributable to TMD.

By carefully framing our findings as associations between anxiety and TMD-like symptoms, our approach offers relevant contributions to understanding early indicators and psychosocial drivers of orofacial symptoms in high-stress populations.

## Materials and Methods

As part of the research, a cross-sectional study design was employed. The Institutional Review Board of King Khalid University’s College of Dentistry granted ethical approval (IRB/KKUCOD/ETH/2022-23/017), and written informed consent was obtained from all participants.

### Participants

This study focused on undergraduate dentistry students at the College of Dentistry, King Khalid University in Aseer Province, Saudi Arabia. A sample size of 83 individuals was determined to achieve a 95% confidence level with a 0.06 margin of error, assuming a 50% prevalence of TMD. Data were collected between December 2022 and June 2023 through voluntary participation. Using convenience sampling, we targeted 200 eligible clinical dental students, inviting them *via* email to participate in an online survey hosted on Survey Monkey (San Mateo, CA). The survey was conducted in English. Of these, 89 students provided informed consent and completed the survey, resulting in a 44.5% response rate.

To ensure awareness and engagement, the study’s objectives, voluntary nature, and confidentiality measures were communicated through in-class announcements and follow-up emails. Participants accessed the survey *via* an introductory page that explained the study’s purpose, the anonymity of data collection, and their right to withdraw at any time. This approach allowed students to complete the survey privately and at their convenience, minimizing potential biases.

### Survey instruments

The Oral Health Impact Profile for Temporomandibular Disorders (OHIP-TMD) was utilized to assess TMD-related impacts on oral health-related quality of life. Scores from the physical function domain were specifically analyzed as indicators of TMD. These scores were derived by summing responses to relevant questions on pain, mobility, and discomfort, with higher scores reflecting more severe impacts. The tool has been validated in previous studies for TMD evaluation (Yule et al., 2015; Durham et al., 2011). For this study, we focused on specific domains such as pain, jaw mobility, and social functioning to assess TMD severity.

The Patient-Reported Outcomes Measurement Information System (PROMIS) has designed a range of available assessments for adults that target bodily, intellectual, and congenial well-being. A worksheet was used to document signs of anxiety. This questionnaire has proven to be a reliable tool for assessing inner consistency, test-retest veracity, and concept viability. Four items were included to assess self-reported levels of fear, anxious-worry, and hyperarousal. Responses are evaluated using a five-point scale ranging from never to always, with scores ranging from 1 to 5. Adding up the responses gives the total score, with higher scores indicating higher levels of anxiety (Reeve et al., 2007).

In our study, we employed both the OHIP and PROMIS survery instruments, each presented in the English language, to guide our investigation.

### Statistical analysis

Demographic information was gathered using eight questions that inquired about age, gender, academic year, employment status, living situation, parental status, relationship status, and recent facial or jaw trauma within the past month.

The collected data from respondents were subjected to statistical treatment using appropriate statistical techniques. The means, SDs, frequencies, and percentages were used as descriptive statistics for summarizing the raw data. Principal component analysis (PCA) is a multidimensional analytical method primarily used for condensing a large number of variables into a few meaningful components without sacrificing much information contained in the original variables of the OHIP questionnaire. PCA attempts to identify the hidden new dimensions in the original variables of OHIP. Before starting PCA, the OHIP data were normalized to reduce the intervariability among variables. A correlation matrix is then generated using the variables, and then the normalized eigenvectors (principal components) are generated. Eigen values greater than or equal to one were retained as the main components that provided the maximum amount of information contained in the original data. The next step involved was to determine the correlation between the principal components and the original variables, which was called factor loading. The popularly known oblique rotation has been used for calculating factor loadings. Factor loadings exceeding 0.4 were used to combine variables to uncover underlying dimensions within the dataset. The subsequent line of evaluation consisted of forging distinctive linear regression models for anxiety and TMD manifestations to measure the linkage among every concept and cohort variable. Finally, a general linear model was applied to evaluate the juxtaposition of anxiety and the psychosocial and functional scales that were included in the principal component analysis of the OHIP-TMD questionnaire. We have assessed the crucial assumptions to be satisfied to perform simple linear modeling and generalized linear modeling. In all the inferential analyses, a calculated *P* value less than 0.05 was considered to indicate statistical significance. All the analyses were carried out with the help of the software SPPS v23.0 for Windows (SPSS, IBM Corp., Armonk, NY, USA).

Factor 1 (items 19–22) related to a construct reflecting psychosocial issues regarding OHRQoL

Factor 2 (items 2–5 and item 7) was related to a construct reflecting physical function issues related to TMD

Factor 3 (items 14–16 and items 12 and 17) related to the construct reflecting social disability

Factor 4 related to a construct reflecting dental issues

Factor 5 related to a construct reflecting eating handicap

## Results

Our study achieved a 44.5% response rate, as 89 out of the 200 invited clinical dental students completed the survey and provided informed consent. Here is the demographic breakdown of the 89 participants: the majority were male (58.4%), with females making up 41.6% of the sample. Regarding academic year distribution, 52.8% were third-year students, 23.6% were in their fourth year, and 16.9% were fifth-year students. Most participants were single (97.8%), with only 2.2% being married. The average ages were 21.66 years for third-year students, 22.51 years for fourth-year students, and 23.47 years for fifth-year students. Table 1 delineates the demographic particulars of the participants, encompassing “gender, marital status, and age distribution”.

**Table 1:** Demographic characteristics of the study cohort. This table presents the demographic details of the study participants, including gender, marital status, and age distribution across academic levels. These demographic variables were analyzed to assess their influence on anxiety (PROMIS scores) and TMD-related impacts (OHIP-TMD scores).

Variable	Category	Third year	Fourth year	Fifth year
Gender	Female	16 (34%)	8 (38.1%)	13 (86.7%)
	Male	31 (66%)	13 (61.9%)	2 (13.3%)
Marital	Single	47 (100%)	21 (100%)	13 (86.7%)
	Married	0 (0%)	0 (0%)	2 (13.3%)
Age	Years	21.66 ± 0.29	22.51 ± 0.34	23.47 ± 0.44

Table 2 presents the loadings of each item for the respective factors. The items were categorized into scales based on their factor loadings. The homogeneity and internal consistency of the scales were assessed using corrected item-total correlations and Cronbach’s alpha, respectively. The corrected item-total correlations ranged from 0.308 to 0.779, as did the Cronbach’s alpha values for psychosocial issues, physical function, and social disability. Dental issues, and eating handicap scales were 0.791, 0.853, 0.921, 0.881 and 0.821, respectively, which indicated acceptable internal consistency.

**Table 2:** Pattern matrix of OHIP-TMD questionnaire principal components. This table displays the principal component analysis (PCA) results of the OHIP-TMD questionnaire, categorizing items into five dimensions: psychosocial issues, physical function, social disability, dental issues, and eating handicap. Factor loadings exceeding 0.4 indicate strong correlations between the items and the identified constructs.

OHIP original variables	Principal components				
	1	2	3	4	5
1. Have you been unable to work to your full capacity?	0.904	0.161	0.072	0.017	0.038
2. Have you been a bit irritable with other people?	0.856	0.287	0.095	−0.052	0.184
3. Has life in general been less satisfying?	0.844	0.055	0.213	0.201	0.122
4. Have you had difficulties doing your usual jobs?	0.817	0.151	0.258	−0.044	0.234
5. Has your concentration been affected?	0.735	0.042	0.470	0.152	0.092
6. Have you had a sore jaw?	0.282	0.767	0.021	0.253	0.096
7. Have you had any painful aching in your mouth?	0.004	0.760	0.018	0.084	0.312
8. Have you had headaches?	0.076	0.669	0.115	0.185	0.186
9. Have you felt that your speech was painful because of problems with your teeth, mouth, dentures or jaws?	0.167	0.667	0.064	0.240	−0.178
10. Have you had difficulties in opening and closing your mouth?	0.142	0.637	0.334	−0.097	0.194
11. Has your sleep been interrupted?	0.153	−0.029	0.827	−0.026	−0.099
12. Have you been upset?	0.133	0.397	0.682	0.357	0.102
13. Has it been difficult to relax?	0.355	0.257	0.672	0.126	0.271
14. Have you felt depressed?	0.594	0.027	0.650	0.008	0.011
15. Have you had to avoid eating some foods?	0.241	0.196	0.472	0.384	0.342
16. Have you been worried by dental problems?	0.029	0.178	0.025	0.776	0.103
17. Have you had dental problems that have made you miserable?	0.106	0.300	0.059	0.764	0.024
18. Have you felt tense because of problems?	0.161	0.172	0.435	0.704	−0.030
19. Have you been self-conscious?	−0.093	−0.028	−0.009	0.532	0.265
20. Have you had difficulties chewing any foods?	0.312	0.154	−0.023	0.163	0.796
21. Has it been uncomfortable to eat any foods?	0.087	0.462	0.069	0.184	0.634
22. Have you had to interrupt meals?	0.292	0.141	0.421	0.142	0.502

**Note:**

Percentage of explained variance 69.413.

The following step in the analysis was to create individual linear regression models for anxiety and TMD symptoms, aiming to investigate the correlation between each variable and demographic factors. All independent variables were simultaneously included in the regression using standard practice. Demographic factors such as age (continuous values), sex (female = 0; male = 1), year of the program (3rd year, 4th year, 5th year), and marital status (0 = single; 1 = unmarried) were entered into the models. Throughout the analysis process, assumptions for regression were evaluated. The Durbin-Watson statistic produced values of 1.999 and 1.998 to check for independence of observations utilizing residuals, while normality of residuals was confirmed with a P-P plot. Additionally, homoscedasticity was validated through a scatter plot of unstandardized residuals, and multicollinearity was assessed by checking variance inflation factor values below 10.

Finally, a general linear model was employed to examine the link between anxiety and the psychosocial and functional scales derived from the principal component analysis of the OHIP-TMD questionnaire. This approach included all factors in the analysis, allowing for adjustment of covariance between dependent variables. It was chosen based on our prior assumption that there would be some level of correlation among the psychosocial and physical function dependent variables.

From Table 3, the mean value for OHIP was 0.60, with an SD of 0.61, indicating that the majority reported never/hardly ever OHIP. The main psychosocial issues were a less satisfied life and difficulty doing their usual jobs. In terms of physical function, the main factors were painful aching of the mouth and sore jaw. The main components of social disability were difficulty relaxing and being upset. Among the dental issues were dental issues and self-concern. Chewing food is a main issue in eating handicaps.

**Table 3:** Prioritization of OHIP-TMD dimensions based on mean scores. This table summarizes the mean and standard deviation (SD) scores for each dimension of the OHIP-TMD questionnaire. The dimensions are categorized into psychosocial issues, physical function, social disability, dental issues, and eating handicap, highlighting the most significant impacts reported by participants.

Factors	OHIP-TMD	Mean	SD
Psychosocial issues	Have you been unable to work to your full capacity?	0.55	1.02
	Have you been a bit irritable with other people?	0.47	0.99
	Has life in general been less satisfying?	0.63	1.08
	Have you had difficulties doing your usual jobs?	0.61	1.11
	Has your concentration been affected?	0.57	1.06
Physical function	Have you had a sore jaw?	0.71	1.10
	Have you had any painful aching in your mouth?	0.72	1.04
	Have you had headaches?	0.53	0.94
	Have you felt that your speech was painful because of problems with your teeth, mouth, dentures or jaws?	0.29	0.77
	Have you had difficulties in opening and closing your mouth?	0.30	0.73
Social disability	Has your sleep been interrupted?	0.33	0.87
	Have you been upset?	0.67	1.09
	Has it been difficult to relax?	0.70	1.12
	Have you felt depressed?	0.54	1.05
	Have you had to avoid eating some foods?	0.61	1.08
Dental issues	Have you been worried by dental problems?	1.51	1.41
	Have you had dental problems that have made you miserable?	1.23	1.36
	Have you felt tense because of problems?	0.76	1.17
	Have you been self-conscious?	2.49	1.42
Eating handicap	Have you had difficulties chewing any foods?	0.45	0.91
	Has it been uncomfortable to eat any foods?	0.29	0.76
	Have you had to interrupt meals?	0.22	0.66
	Overall OHIP-TMD	0.60	0.61

This regression analysis depicted in Table 4 unveils the intricate interplay between demographic variables such as gender, age, and academic level, and OHIP-TMD scores, assessing their impact on the oral health-related quality of life among dental students. Gender emerges as a significant factor, with a negative beta coefficient (−0.268) and a *P*-value of 0.016, illustrating that males exhibit lower OHIP-TMD scores compared to females. This finding suggests that female students endure higher levels of psychosocial or physical impacts on their oral health, likely due to greater susceptibility to stress or anxiety-related symptoms.

**Table 4:** Regression analysis for the OHIP-TMD scores. This table presents the regression analysis of demographic predictors (gender, age, academic level, and marital status) on OHIP-TMD scores. Significant predictors include gender and marital status, with females and married participants reporting higher psychosocial and functional impacts. (*p* < 0.05 indicates statistical significance).

Predictors	Beta	*P*	95% LCL	95% UCL
Gender	−0.268	0.016*	−0.594	−0.063
Age	−0.049	0.659	−0.084	0.054
Level	−0.090	0.443	−0.255	0.113
Marital	0.356	0.001*	0.570	2.264

**Notes:**

R^2^ = 0.200, F(4,78) = 4.847, *P* = 0.002*.

* :*P* < 0.0.

Conversely, age and academic level do not appear as significant predictors, with beta values of −0.049 (*P* = 0.659) and −0.090 (*P* = 0.443), respectively. This indicates that, within the scope of our study, variations in age and academic year do not markedly influence the impact on oral health, implying that the stressors specific to clinical dental studies are uniformly experienced irrespective of these factors.

The overall model, indicated by an R^2^ value of 0.200, signifies that approximately 20% of the variability in OHIP-TMD scores can be explained by these demographic predictors. The significant F-statistic (*P* = 0.002) underscores the model’s statistical relevance, highlighting the pivotal role of gender as a key factor affecting oral health impacts within this cohort. These findings underscore the critical influence of psychosocial stressors and gender-specific responses, with female students being more adversely impacted. This disparity reflects differences in stress management, workload distribution, and the availability of psychosocial support systems.

The regression analysis for PROMIS in Table 5 highlights the nuanced relationships between various demographic predictors and their impact. The analysis illuminates gender as a pivotal factor, with females reporting higher levels of stress or anxiety symptoms, as evidenced by significant differences in PROMIS scores. This underscores the heightened psychosocial burdens carried by female students within the academic environment.

**Table 5:** Regression analysis for PROMIS anxiety scores. This table displays the regression analysis of demographic predictors (gender, age, academic level, and marital status) on PROMIS Anxiety scores. Gender emerged as a significant predictor, with females reporting higher levels of anxiety, indicating a greater vulnerability to psychosocial stressors. (*p* < 0.05 indicates statistical significance).

Predictors	Beta	*P*	95% LCL	95% UCL
Gender	−0.213	0.035*	−3.404	−0.051
Age	0.186	0.115	−0.091	0.828
Level	−0.011	0.933	−1.277	1.173
Marital	0.021	0.851	−5.117	6.184

**Notes:**

R^2^ = 0.092, F(4,78) = 5.691, *P* = 0.048*.

* : *P* < 0.05.

In contrast, age, academic level, and marital status do not significantly influence PROMIS scores, suggesting a uniform experience of stressors irrespective of these demographics. The model’s R^2^ value of 0.092, coupled with a significant F-statistic, confirms the statistical relevance of these findings.

Overall, this analysis accentuates gender-specific responses to stress, signaling the need for tailored interventions to support female students’ well-being in the demanding context of clinical dental education.

The PROMIS Anxiety scores revealed significant correlations with TMD-related impacts, particularly in the psychosocial and physical function domains, as demonstrated in Table 6. Statistical analysis indicated a strong correlation with psychosocial issues (partial η^2^ = 0.801, *P* = 0.003) and a moderate correlation with physical function impairments (partial η^2^ = 0.307, *P* = 0.035). These findings underscore the significant psychosomatic interplay contributing to the severity of TMD symptoms. Higher anxiety levels, as captured by PROMIS, are closely linked with greater psychosocial and physical impairments, reinforcing the role of anxiety as a key driver of TMD-like symptoms in high-stress populations such as clinical dental students.

**Table 6:** Correlation between PROMIS anxiety and OHIP-TMD dimensions. This table demonstrates the correlations between PROMIS Anxiety scores and OHIP-TMD subscales. A strong correlation is observed with psychosocial issues (partial η^2^ = 0.801, *p* = 0.003), and a moderate correlation is noted with physical function (partial η^2^ = 0.307, *p* = 0.035). These results highlight the association between anxiety and TMD-related impairments.

PROMIS	OHIP	*P*	Partial η^2^
Anxiety	Psychosocial issues	0.003*	0.801
	Physical function	0.035*	0.307

**Note:**

* : *P* < 0.05.

A general linear model analysis reported that an increase in anxiety was significantly associated with an increase in psychosocial issues and physical function.

Anxiety was assessed using the PROMIS Anxiety subscale, a validated instrument designed to measure the frequency and severity of anxiety symptoms. Psychosocial issues were evaluated using the OHIP Psychosocial Disability subscale, which captures the impact of oral health on psychological and social aspects of life, including emotional well-being and social interactions. Physical function was measured through the OHIP Physical Disability subscale, assessing physical impairments and functional limitations related to oral health, such as difficulties with eating and speech.

This indicates that higher levels of anxiety are strongly linked with greater psychosocial impacts and moderately linked with physical function impacts, highlighting the profound influence of anxiety on both psychosocial and physical aspects of oral health.

## Discussion

This study bridges a critical gap by examining the link between anxiety and TMD among clinical dental students—a group notably prone to high stress. We aimed to uncover how anxiety interacts with psychosocial and functional impacts of TMD, factoring in demographic variables. The goal was to provide actionable insights for mitigating stress and enhancing student well-being and performance.

It is believed that the Diagnostic Criteria for Temporomandibular Disorders (DC/TMD) is the most accurate approach for TMD diagnosis (Schiffman et al., 2014). A number of research organizations are currently investigating the diagnostic criteria for temporomandibular disorders (DC/TMD) and have discovered that women and younger individuals have a greater prevalence of TMD (Wieckiewicz et al., 2020; Katsikogianni et al., 2020; Bertoli et al., 2018; de Melo Júnior et al., 2019). The prevalence of TMD has been found to be greater among university students, specifically those pursuing medical and dental disciplines, as evidenced by these studies (Wieckiewicz et al., 2020; Katsikogianni et al., 2020; Bertoli et al., 2018; de Melo Júnior et al., 2019).

In a recent study conducted by Srivastava et al. (2021) the prevalence of TMDs was assessed among dental students in Saudi Arabia using a DC/TMD diagnostic tool. The study revealed that dental students, particularly those at clinical levels, were shown to have a greater likelihood of developing TMD (Srivastava et al., 2021). Our study appears to be the only study carried out in the Kingdom of Saudi Arabia utilizing statistical techniques to explore the OHIP-TMD structure. Through these instruments, it was determined that physical and psychosocial difficulties were associated with anxiety among female and married dental students. According to the instrument creators, the questionnaire was thought to include seven areas. Our research revealed that the OHIP-TMD comprises five variables, as illustrated in Table 1. Of all the variables identified, only two had enough elements to create dependable scales. Additional research is needed to improve the psychometric properties of the OHIP-TMD.

The multifactorial analysis of our study revealed that anxiety significantly correlates with increased psychosocial and physical function issues among dental students. This outcome aligns with findings from studies that highlight psychological stress as a major contributor to TMD symptoms. For instance, research by Monteiro et al. (2011) confirmed that university students with higher anxiety levels report more intense orofacial pain associated with TMD, underlining the psychosomatic nature of the disorder. Similarly, Reissmann et al. (2013) demonstrated that a general disposition to anxiety can predispose individuals to TMD-related pain, supporting our results that female students, who reported higher anxiety, experienced greater psychosocial distress.

Our findings also parallel (Boscato et al., 2013) who indicated that anxiety exacerbates the perception of pain and muscular overactivity, critical components in TMD development. While we observed that demographic factors such as sex and marital status influenced the outcomes, this corroborates prior studies like Oliveira et al. (2015) which emphasized that trait anxiety and demographic nuances play roles in TMD severity.

Compared to studies on broader populations, our data specifically emphasize the dental student cohort, which is subject to unique academic and clinical pressures. The consistent association of anxiety with physical and psychosocial issues suggests that interventions focusing on stress management may be essential to improving both academic performance and health outcomes in this high-risk group. Future studies should continue to explore these dynamics with longitudinal designs to establish causality and intervention effectiveness.

The strong correlation between PROMIS Anxiety scores and TMD-related psychosocial and physical impairments highlights the intricate interaction between psychological stress and TMD symptoms. The findings align with prior research that emphasizes the psychosomatic nature of TMD, where heightened anxiety exacerbates muscle tension, pain sensitivity, and functional limitations (Monteiro et al., 2011; Reissmann et al., 2018). Specifically, the strong correlation with psychosocial issues (partial η^2^ = 0.801) reflects the profound psychological distress linked to TMD, while the moderate correlation with physical function (partial η^2^ = 0.307) suggests that anxiety manifests in physical limitations as well. These results advocate for stress-management interventions aimed at mitigating the psychological burden on students, potentially reducing TMD symptoms and improving their overall well-being and academic performance.

Our analysis indicated that the mean OHIP score of 0.60 (SD = 0.61) suggested minimal reported impacts on oral health among the majority of respondents. The overall low impact observed in the OHIP and PROMIS scores for most respondents could be attributed to several potential reasons. First, the study participants, being clinical dental students, might have developed adaptive coping strategies to manage academic stress effectively over time. These students often undergo rigorous training, which may enhance their resilience and familiarity with stressors, diminishing the perceived impact of anxiety and TMD symptoms on their daily functioning.

Second, while anxiety was present, it may not have reached levels severe enough to translate into significant functional impairments. This aligns with research suggesting that mild-to-moderate anxiety may heighten awareness without substantially disrupting quality of life. Additionally, the supportive academic environment, including access to mental health resources and peer support, could contribute to mitigating the effects of anxiety on oral health and functional status.

Although there is limited research specifically on Aseer province, general studies in rural or less densely populated areas have reported lower levels of stress and anxiety compared to urban centers. For instance, studies in other rural regions highlight that lifestyle factors, slower pace of life, and environmental influences can contribute to lower stress levels and greater psychological well-being. Research such as that by Staniszewski et al. (2018) which explores stress-related impacts and cortisol levels, indicates that environment and daily life stressors play significant roles in psychological health and TMD development.

In a recent systematic evaluation, the effectiveness of psychological interventions for alleviating anxiety among university students was examined. According to the study, anxiety levels decreased significantly in response to cognitive, behavioral, and mindfulness interventions. Research suggests that universities should provide interventions for their students, given the correlation between elevated levels of anxiety and diminished academic performance, increased suicidal ideation, and decreased engagement (Regehr, Glancy & Pitts, 2013). In clinical settings, students frequently encounter distress as a result of apprehension regarding their aptitude for patient management. By instituting routine evaluation and direction at the unit level, this concern might be mitigated.

The findings of this study underscore the importance of exploring psychosocial factors influencing TMD from multiple perspectives. Psychological dimensions such as coping mechanisms, individual resilience, and the unique academic pressures faced by dental students play a pivotal role in the manifestation and severity of TMD symptoms. Understanding these dynamics can offer deeper insights into the biopsychosocial model of TMD.

Targeted interventions that address stress management—such as mindfulness training, cognitive-behavioral therapy (CBT), and resilience-building programs—may mitigate anxiety and its associated impacts on TMD symptoms. These strategies could be implemented within academic institutions to support students’ mental health and overall well-being, potentially reducing the burden of TMD-related discomfort.

To further validate these findings, future research should employ longitudinal study designs to track the progression of TMD symptoms over time and their relationship with stress and anxiety. Additionally, expanding the study to include diverse populations beyond clinical dental students would enhance the generalizability of the results. Such efforts could reveal broader patterns and provide a more comprehensive understanding of the psychosocial drivers of TMD.

A key limitation of this study is the reliance on self-reported measures for assessing TMD symptoms, which may introduce subjective bias. While validated tools such as the OHIP-TMD and PROMIS instruments were utilized to ensure reliability and consistency, the absence of clinical validation limits the ability to confirm TMD diagnoses definitively. Clinical examinations following standardized diagnostic criteria, such as the DC/TMD (Diagnostic Criteria for Temporomandibular Disorders), should be incorporated into future research to corroborate self-reported findings.

Another limitation is the exclusive focus on clinical dental students, a population known to experience high levels of stress and anxiety. While this group provides valuable insights into the interplay between psychosocial stressors and TMD symptoms, the findings may not be generalizable to other populations with differing stress profiles or demographic characteristics. Future studies should consider including broader and more diverse participant cohorts to enhance generalizability and explore these associations across different environments and cultural contexts.

## Conclusions

The findings of our research indicate that the majority of clinical students did not perceive any impact on their OHRQoL. However, there was a significant correlation between anxiety and both oral physical function impairment and psychosocial distress in specific subsets of female students. Moreover, anxiety affects not only the quality of life associated with oral health but also the engagement of students in the learning process and their academic performance. Given the anxiety levels documented among this cohort of students, the implementation of anxiety reduction interventions might prove beneficial in potentially mitigating symptoms associated with the temporomandibular joint.

## Supplemental Information

10.7717/peerj.19066/supp-1 Supplemental Information 1 Raw data.

## Additional Information and Declarations

### Data Availability

The following information was supplied regarding data availability:

The raw data is available in the Supplemental Files.
